# Supplementary material for: Swin Transformer Improves the IDH Mutation Status Prediction of Gliomas Free of MRI-Based Tumor Segmentation
Source: J Clin Med. 2022 Aug 8;11(15):4625. doi: 10.3390/jcm11154625 (PMC9369996; doi:10.3390/jcm11154625)
Supplement: Supplementary file 1 [file jcm-11-04625-s001.zip › jcm-1836023-supplementary.pdf]

Supplementary Materials

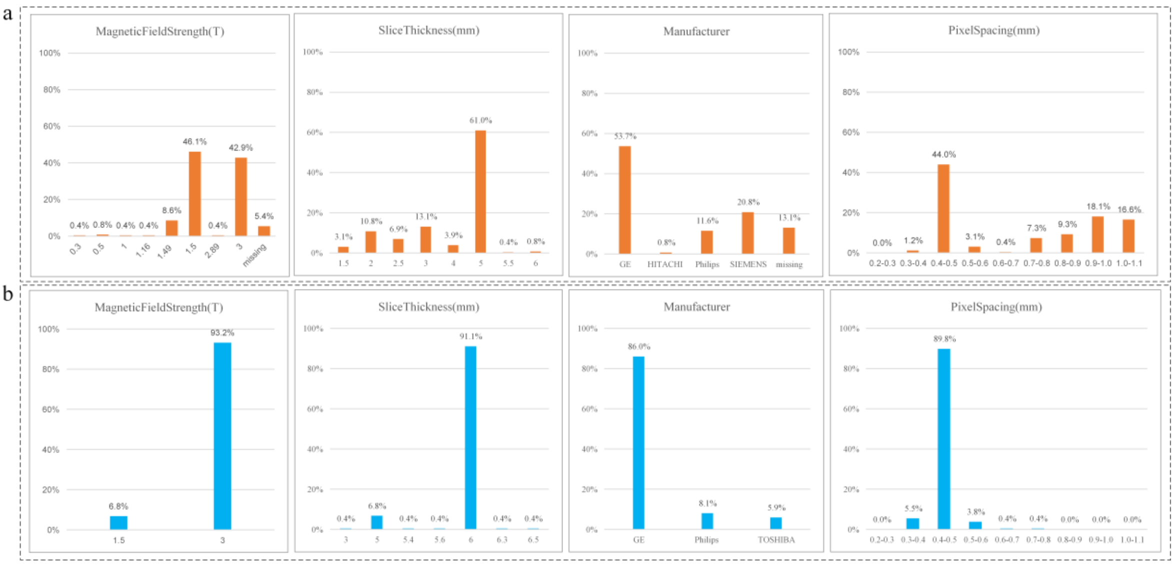

Figure S1. Image Acquisition Parameters. (**a**) Image Acquisition Parameters of TCIA; (**b**) Image Acquisition Parameters of AHXZ.

As showed in the above eFigure 1, 3T in the TCIA (42.9%) and in the AHXZ (93.2%), 1.5T in the TCIA (46.1%) and in the AHXZ (6.8%). SliceThickness: 5 mm in the TCIA (61.0%) and 6mm in the AHXZ (91.1%). Manufacturer: GE in the TCIA (53.7%) and in the AHXZ (86.0%), Philips in the TCIA (11.6%) and in the AHXZ (8.1%), SIMENS only in TCIA (20.8%). PixelSpacing:0.4-0.5mm in the TCIA (44.0%) and in the AHXZ (89.8%).

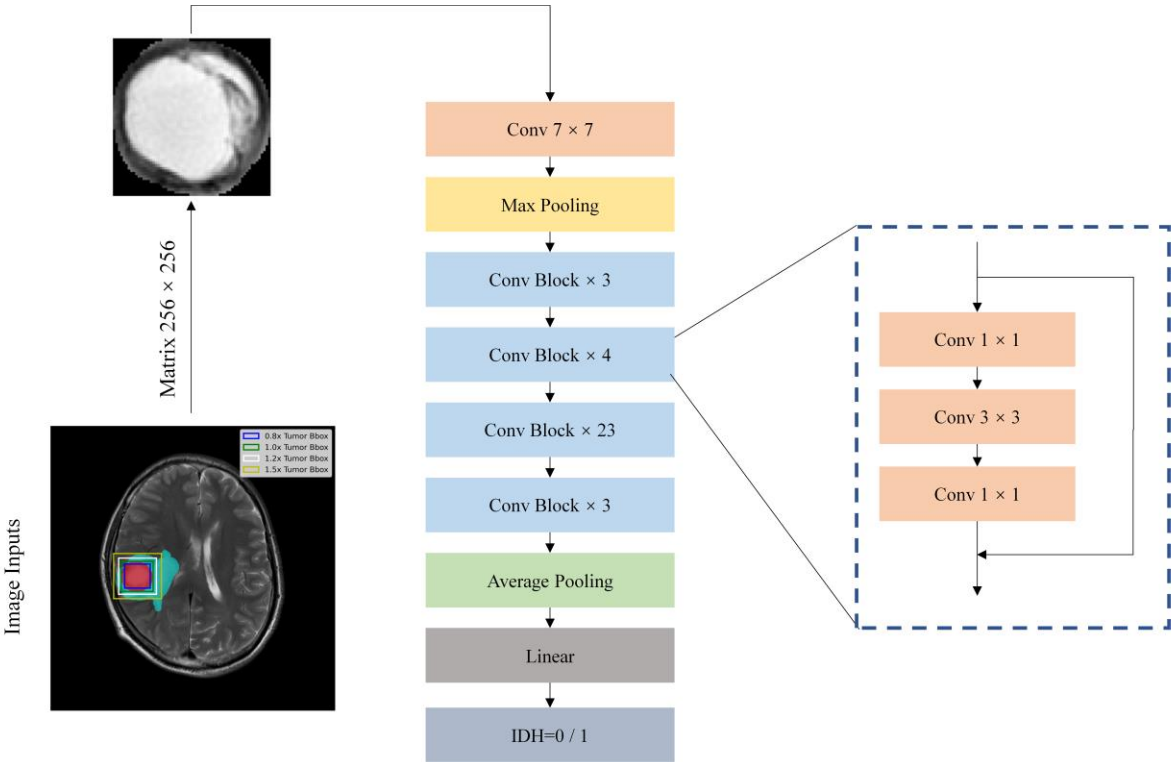

Figure S2. ResNet architecture.

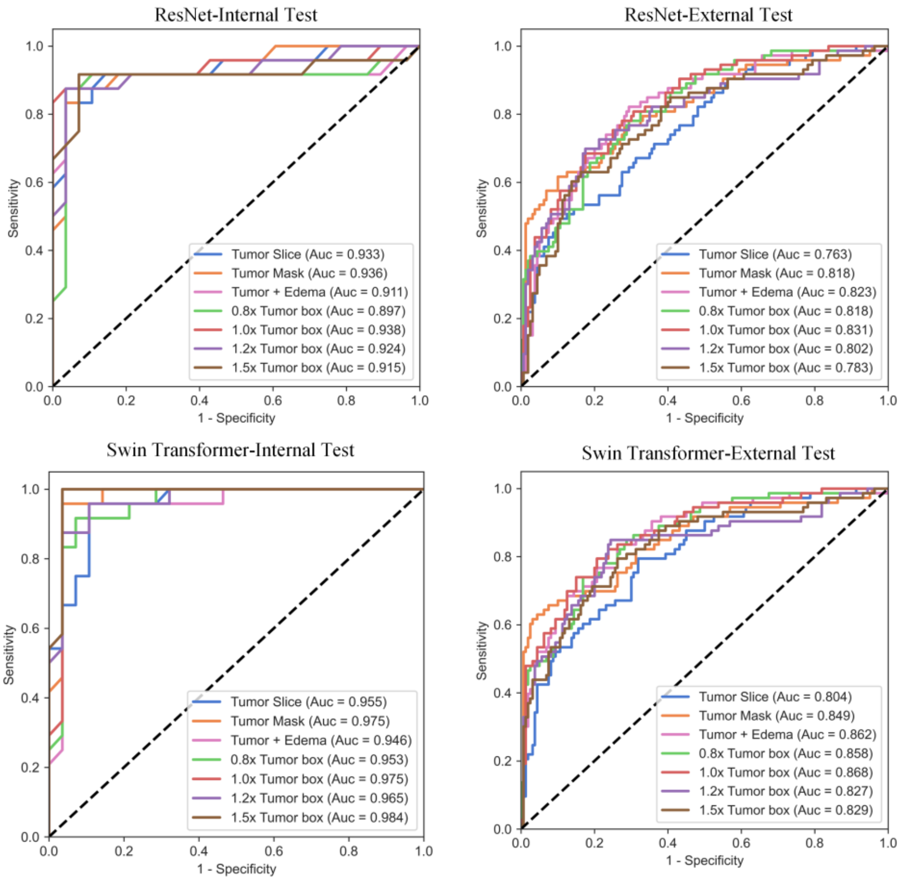

Figure S3. ROCs of all the image-based models.

Imaging Preprocessing and Augmentation

All T2 images were preprocessed sequentially: (1) N4BiasCorrection to remove radiofrequency inhomogeneity using SimpleITK package (https://simpleitk.org, accessed on 1 May 2021). (ii) intensity normalization to zero mean and unit variance using MONAI project (https://docs.monai.io/en/latest/index.html). The images were cropped using the intensity threshold of 150 to generate brain only images, and the intensity was normalized to [0,1] by subtracting the mean value and dividing the standard deviation of the whole volume signal intensity. (iii) selecting the slices that involved the tumor region. The first slice on two sides was discarded in order to prevent the interference of slices which only contain a small area of tumor. (iv) resampling to sizes of 256 × 256 and expanding to 3 channels by simply repeat the first channel.

We leverage the following data augmentations during the training for both Swin Transformer and ResNet to improve the model generalization ability: geometric transformations (i.e., random flip and random rotate) and intensity transformations (i.e., random adjust contrast, random gaussian noise and random gaussian smooth). Empirically, augmentations facilitate to improve the model ability of learning geometric invariant features and invariance to noise. The probability of flipping and rotate were set to 0.5, the probability of random adjust contrast was set to 0.1 and the gamma coefficient is set to (0.5, 4.5). The mean of gaussian noise is zero and the standard deviation is 0.1. The parameters of gaussian smooth is: sigma_x = (0.25, 1.5), sigma_y = (0.25, 1.5).

Additionally, all the axial slices per patient were automatically selected based on tumor segmentation or box, and were considered as individual samples in model development and testing. In other worlds, the training and inference are presented on slice-level. We assumed that with the intra patient heterogeneity extracting multiple images per patient provide a data augmentation effect beyond artificial data augmentation such as flipping or rotation.

The Description of Swin Transformer Block

Swin Transformer block consisting of two successive blocks and composed of four-layer normalization (LN) layers, a window multi-head self-attention (W-MSA), two multilayer perceptrons (MLP) and a shifted-window multi-head self-attention (SW-MSA). Instead of using multi-head self-attention (MSA), Swin Transformer used W-MSA and SW-MSA followed by an MLP. In particular, a LN is introduced before each MSA module and MLP, and a residual connection is conducted after each module. The shifted windowing scheme brings greater efficiency by limiting self-attention computation to nonoverlapping local windows while also allowing for cross-window connection. Because transformer-based model needs a large volume of data to train, and thus is difficult to train, we used ImageNet pretrained weights as the model initialization.

The CNN-Based ResNet Network Development

ResNet contains the initial 7 × 7 convolution and max-pooling, and layers 1–4 comprising 3, 4, 23, and 3 residual blocks with each residual block having one 3 × 3 convolution followed by 1 × 1 convolution followed by 3 × 3 convolution. Then, the output of the 4-th layer is applied to an average pooling layer and a 2-d fully connected layer. The final prediction is computed by applying a softmax activation function on the output of fully connected layer.

Hybrid Model Development

For age information, we directly encode the numeric value of age into a vector with only one value. For lesion location information, we encode the position information into a one-hot vector with a length of 6. Then, we concatenated these two vectors to the feature vector before "fc" layer in ResNet and feature vector before "classification head" module in Swin Transformer. In the training stage, we frozen weights of all the layers except "fc" in ResNet and "classification head" in Swin Transformer, and re-train these two layers to integrate extra age and lesion location information. Since the weights of the origin model is frozen, integrating age and lesion location information will not influence the performance of origin model.

Table S1. List of the enrolled patients from TCIA set.

SUBJECT ID	Age	Gender	Histology	Grade	IDH (WT = 0, Mutant = 1)	Survival (Months)	Vital (0 = alive 1 = death)	Days to Death or Alive	Recurrence or Progression after Initial Treatment (Days)
TCGA-02-0003	50	male	glioblastoma	G4	WT	4.7311	1	144	40

TCGA-02-0006	56	female	glioblastoma	G4	WT	18.333	1	558	302
TCGA-02-0009	61	female	glioblastoma	G4	WT	10.5793	1	322	264
TCGA-02-0011	18	female	glioblastoma	G4	WT	20.6986	1	630	145
TCGA-02-0027	33	female	glioblastoma	G4	WT	12.1563	1	370	257
TCGA-02-0033	54	male	glioblastoma	G4	WT	2.8255	1	86	32
TCGA-02-0034	60	male	glioblastoma	G4	WT	14.1276	1	430	386
TCGA-02-0037	74	female	glioblastoma	G4	WT	3.614	1	110	37
TCGA-02-0046	61	male	glioblastoma	G4	WT	6.8667	1	209	195
TCGA-02-0047	78	male	glioblastoma	G4	WT	14.719	1	448	57
TCGA-02-0054	44	female	glioblastoma	G4	WT	6.5381	1	199	72
TCGA-02-0060	66	female	glioblastoma	G4	WT	6.0124	1	183	183
TCGA-02-0064	50	male	glioblastoma	G4	WT	19.7129	1	600	496
TCGA-02-0069	31	female	glioblastoma	G4	WT	28.6823	0	873	873
TCGA-02-0075	63	male	glioblastoma	G4	WT	20.83	1	634	336
TCGA-02-0085	63	female	glioblastoma	G4	WT	51.2865	1	1561	976
TCGA-02-0086	45	female	glioblastoma	G4	WT	8.8051	1	268	97
TCGA-02-0102	42	male	glioblastoma	G4	WT	27.0067	1	822	450
TCGA-06-0119	81	female	glioblastoma	G4	WT	2.6941	1	82	NA
TCGA-06-0122	84	female	glioblastoma	G4	WT	6.1439	1	187	NA
TCGA-06-0128	66	male	glioblastoma	G4	Mutant	22.7027	1	691	190
TCGA-06-0129	30	male	glioblastoma	G4	Mutant	33.6434	1	1024	148
TCGA-06-0133	64	male	glioblastoma	G4	WT	14.2919	1	435	78
TCGA-06-0137	63	female	glioblastoma	G4	WT	26.6782	1	812	487
TCGA-06-0139	40	male	glioblastoma	G4	WT	11.8935	1	362	152
TCGA-06-0142	81	male	glioblastoma	G4	WT	2.2013	1	67	NA
TCGA-06-0143	58	male	glioblastoma	G4	WT	11.7292	1	357	265
TCGA-06-0145	53	female	glioblastoma	G4	WT	2.3327	1	71	NA
TCGA-06-0147	51	female	glioblastoma	G4	WT	17.7745	1	541	92
TCGA-06-0148	76	male	glioblastoma	G4	WT	10.0865	1	307	189
TCGA-06-0190	62	male	glioblastoma	G4	WT	10.415	1	317	88
TCGA-06-1806	47	male	glioblastoma	G4	WT	15.3104	1	466	NA
TCGA-06-2570	21	female	glioblastoma	G4	Mutant	31.4959	0	958	NA
TCGA-06-5408	54	female	glioblastoma	G4	WT	11.7292	1	357	158
TCGA-06-5412	78	female	glioblastoma	G4	WT	4.534	1	138	88
TCGA-06-5413	67	male	glioblastoma	G4	WT	8.8051	0	268	195
TCGA-06-5417	45	female	glioblastoma	G4	Mutant	5.0925	0	155	NA
TCGA-06-6389	49	female	glioblastoma	G4	Mutant	7.7866	0	237	NA
TCGA-12-0829	75	male	glioblastoma	G4	WT	20.5672	1	626	473
TCGA-12-1093	66	female	glioblastoma	G4	WT	15.9675	1	486	322
TCGA-12-1598	75	female	glioblastoma	G4	WT	15.6389	1	476	NA
TCGA-12-1601	NaN	NA	glioblastoma	G4	WT	NA	NA	NA	NA
TCGA-12-1602	58	male	glioblastoma	G4	WT	6.7681	1	206	140
TCGA-12-3650	46	male	glioblastoma	G4	WT	10.9407	1	333	NA
TCGA-14-0789	54	male	glioblastoma	G4	WT	11.2364	1	342	105
TCGA-14-1456	23	male	glioblastoma	G4	Mutant	40.9372	0	1246	98
TCGA-14-1794	59	male	glioblastoma	G4	WT	0.98565	1	30	NA
TCGA-14-1829	57	male	glioblastoma	G4	WT	7.1624	0	218	NA
TCGA-14-3477	38	female	glioblastoma	G4	WT	3.7783	0	115	NA
TCGA-19-1390	63	female	glioblastoma	G4	WT	25.364	1	772	NA
TCGA-19-1789	69	female	glioblastoma	G4	WT	3.2526	1	99	NA
TCGA-19-1791	82	female	glioblastoma	G4	WT	0.1314	0	NA	NA
TCGA-19-2620	70	male	glioblastoma	G4	WT	4.8625	1	148	NA
TCGA-19-2624	51	male	glioblastoma	G4	WT	0.16427	1	5	NA

TCGA-19-2631	74	female	glioblastoma	G4	WT	6.9981	1	213	NA
TCGA-19-5953	58	male	glioblastoma	G4	WT	4.7311	1	144	119
TCGA-19-5954	72	female	glioblastoma	G4	WT	12.0987	1	368	NA
TCGA-19-5958	56	male	glioblastoma	G4	WT	14.0712	1	428	356
TCGA-19-5960	56	male	glioblastoma	G4	WT	5.4247	1	455	382
TCGA-27-1830	57	male	glioblastoma	G4	WT	5.0596	1	154	124
TCGA-27-1835	53	female	glioblastoma	G4	WT	21.29	1	648	157
TCGA-27-1838	59	female	glioblastoma	G4	WT	11.4992	1	350	119
TCGA-76-4926	68	male	glioblastoma	G4	WT	4.534	1	138	34
TCGA-76-4927	58	male	glioblastoma	G4	WT	17.5774	1	535	416
TCGA-76-4928	85	female	glioblastoma	G4	WT	3.0884	1	94	NA
TCGA-76-4929	76	female	glioblastoma	G4	WT	3.6469	1	111	NA
TCGA-76-4931	70	female	glioblastoma	G4	WT	9.1665	1	279	112
TCGA-76-4932	50	female	glioblastoma	G4	WT	47.9024	1	1458	NA
TCGA-76-4934	66	female	glioblastoma	G4	WT	2.5298	1	77	NA
TCGA-76-4935	52	female	glioblastoma	G4	WT	10.7764	0	328	NA
TCGA-76-6191	57	male	glioblastoma	G4	WT	16.6903	1	508	323
TCGA-76-6192	74	male	glioblastoma	G4	WT	3.2855	1	100	76
TCGA-76-6193	78	male	glioblastoma	G4	WT	2.6941	1	82	64
TCGA-76-6280	57	male	glioblastoma	G4	WT	11.3678	1	346	108
TCGA-76-6282	63	male	glioblastoma	G4	WT	17.0517	1	519	458
TCGA-76-6285	64	female	glioblastoma	G4	WT	8.3451	1	254	NA
TCGA-76-6286	60	male	glioblastoma	G4	WT	20.9614	1	638	180
TCGA-76-6656	66	male	glioblastoma	G4	WT	4.8297	1	147	NA
TCGA-76-6657	74	male	glioblastoma	G4	WT	5.0268	1	153	135
TCGA-76-6661	54	male	glioblastoma	G4	WT	23.9013	1	727	NA
TCGA-76-6662	58	male	glioblastoma	G4	WT	34.4548	1	1048	205
TCGA-76-6663	44	female	glioblastoma	G4	WT	18.6411	1	567	NA
TCGA-76-6664	49	female	glioblastoma	G4	WT	36.1972	0	1101	NA
TCGA-CS-4938	31	female	astrocytoma	G2	Mutant	117.5014	0	3574	NA
TCGA-CS-4941	67	male	astrocytoma	G3	WT	7.688	1	234	9
TCGA-CS-4942	44	female	astrocytoma	G3	Mutant	43.8613	1	1335	1184
TCGA-CS-4943	37	male	astrocytoma	G3	Mutant	36.3616	1	1106	NA
TCGA-CS-4944	50	male	astrocytoma	G2	Mutant	60.0986	0	1828	no
TCGA-CS-5390	47	female	oligodendroglioma	G2	Mutant	64.5927	1	775	NA
TCGA-CS-5393	39	male	astrocytoma	G3	Mutant	40.1487	0	1222	no
TCGA-CS-5394	40	male	astrocytoma	G3	Mutant	0.0986	0	8	NA
TCGA-CS-5395	43	male	oligodendroglioma	G2	WT	20.9943	1	639	287
TCGA-CS-5396	53	female	oligodendroglioma	G3	Mutant	53.6219	0	1631	no
TCGA-CS-5397	54	female	astrocytoma	G3	WT	6.3739	1	194	no
TCGA-CS-6186	58	male	oligoastrocytoma	G3	WT	17.6759	1	538	188
TCGA-CS-6188	48	male	astrocytoma	G3	WT	26.7616	1	814	647
TCGA-CS-6290	31	male	astrocytoma	G3	Mutant	37.3808	1	1137	no
TCGA-CS-6665	51	female	astrocytoma	G3	Mutant	51.5507	0	1568	no
TCGA-CS-6666	22	male	astrocytoma	G3	Mutant	46.9479	0	1428	no
TCGA-CS-6667	39	female	astrocytoma	G2	Mutant	48.2959	0	1469	no
TCGA-CS-6668	57	female	oligodendroglioma	G2	Mutant	49.9397	0	1519	no

TCGA-CS-6669	26	female	oligodendroglioma	G2	WT	49.1178	0	1494	no
TCGA-DU-5849	48	male	oligodendroglioma	G2	Mutant	14.5547	0	443	no
TCGA-DU-5851	40	female	oligoastrocytoma	G3	Mutant	17.446	0	531	no
TCGA-DU-5852	61	female	oligoastrocytoma	G3	WT	6.7353	1	205	24
TCGA-DU-5853	29	male	oligoastrocytoma	G2	Mutant	13.3719	0	407	no
TCGA-DU-5854	57	female	astrocytoma	G3	WT	8.4437	0	257	202
TCGA-DU-5855	49	female	oligoastrocytoma	G3	Mutant	6.801	0	207	no
TCGA-DU-5871	37	female	oligoastrocytoma	G2	Mutant	18.9244	0	576	no
TCGA-DU-5872	43	female	oligoastrocytoma	G2	Mutant	17.4788	0	532	265
TCGA-DU-5874	62	female	oligodendroglioma	G2	Mutant	15.1461	0	461	no
TCGA-DU-6395	31	male	oligoastrocytoma	G2	Mutant	48.9867	1	1491	1197
TCGA-DU-6399	54	male	oligodendroglioma	G2	Mutant	65.7098	1	2000	1629
TCGA-DU-6400	66	female	oligodendroglioma	G2	Mutant	1.2156	1	37	no
TCGA-DU-6401	31	female	oligodendroglioma	G2	Mutant	87.394	1	2660	1886
TCGA-DU-6404	24	female	oligodendroglioma	G3	WT	133.6537	1	4068	1154
TCGA-DU-6405	51	female	astrocytoma	G3	WT	19.8772	1	605	486
TCGA-DU-6407	35	female	oligodendroglioma	G2	Mutant	94.4578	1	2875	2397
TCGA-DU-6408	23	female	oligodendroglioma	G3	Mutant	114.0065	1	3470	2097
TCGA-DU-7008	41	female	oligodendroglioma	G2	Mutant	156.1265	0	4752	330
TCGA-DU-7010	58	female	astrocytoma	G3	Mutant	14.9818	1	456	193
TCGA-DU-7013	59	male	Astrocytoma	G3	WT	8.8438	1	269	187
TCGA-DU-7015	41	female	oligodendroglioma	G2	Mutant	90.7124	0	2761	591
TCGA-DU-7018	57	female	oligodendroglioma	G3	Mutant	30.6536	1	933	338
TCGA-DU-7019	39	male	oligoastrocytoma	G3	Mutant	26.2839	0	800	no
TCGA-DU-7294	53	female	oligodendroglioma	G2	Mutant	94.2607	0	2869	no
TCGA-DU-7298	38	female	astrocytoma	G3	Mutant	18.9244	1	576	200
TCGA-DU-7299	33	male	astrocytoma	G3	Mutant	43.9927	1	1339	675
TCGA-DU-7300	53	female	oligodendroglioma	G3	Mutant	61.9643	1	1886	317
TCGA-DU-7301	53	male	oligodendroglioma	G2	Mutant	25.8897	1	788	366

TCGA-DU-7302	48	female	oligodendroglioma	G3	Mutant	60.2559	0	1834	1374
TCGA-DU-7304	43	male	oligoastrocytoma	G3	Mutant	23.2941	1	709	309
TCGA-DU-7306	67	male	oligoastrocytoma	G2	Mutant	41.9557	0	1277	1250
TCGA-DU-7309	41	female	oligodendroglioma	G3	Mutant	2.7598	0	84	no
TCGA-DU-8158	57	female	astrocytoma	G3	WT	5.0925	1	155	NA
TCGA-DU-8162	61	female	oligoastrocytoma	G3	WT	14.5876	1	444	402
TCGA-DU-8164	51	male	oligodendroglioma	G2	Mutant	21.3885	0	651	no
TCGA-DU-8165	60	female	oligodendroglioma	G3	WT	19.1216	0	582	no
TCGA-DU-8166	29	female	oligoastrocytoma	G2	Mutant	16.9531	0	516	193
TCGA-DU-8167	69	female	oligoastrocytoma	G2	Mutant	15.4747	0	471	no
TCGA-DU-8168	55	female	oligodendroglioma	G3	Mutant	14.1605	0	431	no
TCGA-DU-A5TP	33	male	astrocytoma	G3	Mutant	33.2712	0	1012	203
TCGA-DU-A5TR	51	male	oligoastrocytoma	G2	Mutant	29.852	0	908	848
TCGA-DU-A5TS	42	male	oligodendroglioma	G2	Mutant	31.6931	0	964	551
TCGA-DU-A5TT	70	male	oligodendroglioma	G3	WT	24.4274	0	743	584
TCGA-DU-A5TU	62	female	astrocytoma	G2	Mutant	26.1698	0	796	186
TCGA-DU-A5TW	33	female	astrocytoma	G3	Mutant	24.9863	0	760	no
TCGA-DU-A5TY	46	female	astrocytoma	G3	WT	33.9616	1	1033	526
TCGA-DU-A6S2	37	female	oligodendroglioma	G2	Mutant	25.5452	0	777	no
TCGA-DU-A6S3	60	male	oligodendroglioma	G2	Mutant	21.5671	0	656	no
TCGA-DU-A6S6	35	female	oligoastrocytoma	G2	Mutant	95.1123	0	2893	2277
TCGA-DU-A6S7	27	female	astrocytoma	G3	Mutant	20.9753	0	638	no
TCGA-DU-A6S8	74	female	oligodendroglioma	G3	Mutant	22.2907	0	678	641
TCGA-FG-5963	23	male	astrocytoma	G3	WT	25.4625	1	775	497
TCGA-FG-5964	62	male	oligodendroglioma	G2	Mutant	52.2082	0	1588	no
TCGA-FG-6688	59	female	astrocytoma	G3	WT	18.7601	0	571	405
TCGA-FG-6689	30	male	astrocytoma	G2	Mutant	14.9161	0	454	218
TCGA-FG-6690	70	male	oligodendroglioma	G2	Mutant	42.5425	0	1294	no
TCGA-FG-6691	23	female	astrocytoma	G2	Mutant	41.326	0	1257	no

TCGA-FG-6692	63	male	oligodendroglioma	G3	WT	18.4316	1	561	no
TCGA-FG-7634	28	male	oligodendroglioma	G2	Mutant	15.3432	0	467	no
TCGA-FG-7637	49	male	oligodendroglioma	G2	Mutant	40.0501	0	1219	no
TCGA-FG-8189	33	female	oligodendroglioma	G2	Mutant	22.5205	0	685	no
TCGA-FG-A4MT	27	female	oligodendroglioma	G2	Mutant	38.2431	0	1164	499
TCGA-FG-A4MU	58	male	Oligoastrocytoma	G3	WT	10.7178	0	326	NA
TCGA-FG-A6IZ	60	male	oligodendroglioma	G2	Mutant	15.0247	0	457	no
TCGA-FG-A6J1	44	female	Oligodendroglioma	G2	Mutant	16.4384	0	500	no
TCGA-FG-A713	74	female	oligoastrocytoma	G2	Mutant	20.4493	0	622	no
TCGA-FG-A87N	37	female	Astrocytoma	G3	Mutant	18.6411	0	567	no
TCGA-HT-7468	30	male	oligoastrocytoma	G3	Mutant	6.6695	0	203	no
TCGA-HT-7469	30	male	oligoastrocytoma	G3	WT	11.5321	1	351	92
TCGA-HT-7471	37	female	oligoastrocytoma	G3	Mutant	0.1314	0	4	NA
TCGA-HT-7472	38	male	oligoastrocytoma	G2	Mutant	0.0329	1	1	no
TCGA-HT-7473	28	male	oligoastrocytoma	G2	Mutant	16.526	0	503	no
TCGA-HT-7475	67	male	oligoastrocytoma	G3	Mutant	17.4131	0	530	no
TCGA-HT-7476	26	Male	astrocytoma	G2	Mutant	6.5381	0	199	no
TCGA-HT-7478	36	Male	astrocytoma	G2	Mutant	6.3739	0	194	no
TCGA-HT-7481	39	Male	oligoastrocytoma	G2	Mutant	59.1388	0	2018	3071
TCGA-HT-7602	21	male	oligodendroglioma	G2	Mutant	29.8322	0	908	NA
TCGA-HT-7603	29	Male	oligodendroglioma	G2	Mutant	23.1627	0	705	no
TCGA-HT-7605	38	male	oligodendroglioma	G2	Mutant	4.5668	0	139	no
TCGA-HT-7606	30	female	oligodendroglioma	G2	Mutant	17.2817	0	526	no
TCGA-HT-7608	61	male	oligoastrocytoma	G2	Mutant	22.0456	0	671	no
TCGA-HT-7616	75	male	oligodendroglioma	G3	Mutant	0.22998	1	7	no
TCGA-HT-7680	32	female	astrocytoma	G2	WT	0.75566	0	23	no
TCGA-HT-7684	58	male	Oligoastrocytoma	G3	Mutant	6.0493	0	184	no
TCGA-HT-7686	29	female	astrocytoma	G3	Mutant	42.7114	0	1300	no

TCGA-HT-7690	29	male	oligoastrocytoma	G3	Mutant	0.098565	0	3	no
TCGA-HT-7692	43	male	oligoastrocytoma	G2	Mutant	2.9569	0	90	no
TCGA-HT-7693	51	female	oligodendroglioma	G2	Mutant	17.5117	0	533	no
TCGA-HT-7694	60	male	oligodendroglioma	G3	Mutant	6.8995	0	210	no
TCGA-HT-7695	29	female	oligodendroglioma	G2	Mutant	14.5219	0	442	no
TCGA-HT-7854	62	Male	astrocytoma	G2	WT	39.4587	0	1201	1147
TCGA-HT-7855	39	male	astrocytoma	G3	Mutant	19.2201	0	585	no
TCGA-HT-7856	35	male	oligodendroglioma	G3	Mutant	39.0645	0	1189	no
TCGA-HT-7860	60	female	astrocytoma	G3	WT	0.49282	0	15	NA
TCGA-HT-7874	41	female	oligodendroglioma	G3	Mutant	37.126	0	1130	no
TCGA-HT-7877	20	female	Oligoastrocytoma	G2	Mutant	0.1315	0	4	no
TCGA-HT-7879	31	male	oligoastrocytoma	G3	Mutant	3.6797	0	112	no
TCGA-HT-7880	30	Male	oligodendroglioma	G2	Mutant	5.3225	0	162	no
TCGA-HT-7882	66	male	oligodendroglioma	G3	WT	3.7126	1	113	no
TCGA-HT-7884	44	female	astrocytoma	G2	Mutant	11.2692	0	343	no
TCGA-HT-7902	30	female	oligodendroglioma	G2	Mutant	31.4093	0	956	no
TCGA-HT-8010	64	female	oligodendroglioma	G2	Mutant	1.6427	0	50	no
TCGA-HT-8013	37	female	oligodendroglioma	G2	Mutant	63.5085	1	1933	1306
TCGA-HT-8018	40	female	oligoastrocytoma	G2	Mutant	37.8739	1	1152	no
TCGA-HT-8019	34	female	oligodendroglioma	G3	WT	32.9863	0	1004	no
TCGA-HT-8105	54	male	oligodendroglioma	G3	Mutant	6.2424	0	190	no
TCGA-HT-8106	53	male	astrocytoma	G3	Mutant	0.098565	0	3	no
TCGA-HT-8107	62	male	oligodendroglioma	G2	WT	0.45997	0	14	no
TCGA-HT-8111	32	male	oligoastrocytoma	G3	Mutant	0.22998	0	7	NA
TCGA-HT-8113	49	female	oligodendroglioma	G2	Mutant	29.5694	0	900	no
TCGA-HT-8114	36	male	oligoastrocytoma	G3	Mutant	34.1917	0	1040	no
TCGA-HT-8563	30	female	astrocytoma	G3	Mutant	28.274	0	860	no
TCGA-HT-A5RC	70	female	astrocytoma	G3	WT	5.3225	1	162	NA
TCGA-HT-A61A	20	female	oligodendroglioma	G2	Mutant	6.3739	0	194	NA
TCGA-02-0048	80	male	glioblastoma	G4	WT	3.2198	1	98	23

TCGA-02-0068	57	male	glioblastoma	G4	WT	26.4153	1	804	175
TCGA-02-0070	70	male	glioblastoma	G4	WT	25.0354	0	762	762
TCGA-06-0127	67	male	glioblastoma	G4	WT	3.9754	1	121	91
TCGA-06-0132	49	male	glioblastoma	G4	WT	25.3311	1	771	482
TCGA-06-0138	43	male	glioblastoma	G4	WT	24.2141	1	737	394
TCGA-06-0154	54	male	glioblastoma	G4	WT	13.9305	1	424	208
TCGA-06-0158	73	male	glioblastoma	G4	WT	10.8093	1	329	90
TCGA-06-0166	51	male	glioblastoma	G4	WT	5.8482	1	178	66
TCGA-06-0168	59	female	glioblastoma	G4	WT	19.6472	1	598	461
TCGA-06-0174	54	male	glioblastoma	G4	WT	3.2198	1	98	47
TCGA-06-0176	34	male	glioblastoma	G4	WT	91.0027	0	2768	41
TCGA-06-0184	63	male	glioblastoma	G4	WT	69.8959	1	2126	1276
TCGA-06-0185	54	male	glioblastoma	G4	WT	73.8411	0	2246	711
TCGA-06-0187	69	male	glioblastoma	G4	WT	27.2039	1	828	531
TCGA-06-0188	71	male	glioblastoma	G4	WT	44.5808	1	1356	310
TCGA-06-0189	55	male	glioblastoma	G4	WT	15.4089	1	469	NA
TCGA-06-0192	58	male	glioblastoma	G4	WT	38.959	1	1185	648
TCGA-06-0213	55	female	glioblastoma	G4	WT	0.52568	1	16	NA
TCGA-06-0237	75	female	glioblastoma	G4	WT	13.6348	1	415	NA
TCGA-06-0238	46	male	glioblastoma	G4	WT	13.3062	1	405	311
TCGA-06-0241	65	female	glioblastoma	G4	WT	48.4904	1	1481	196
TCGA-06-0644	71	male	glioblastoma	G4	WT	12.6247	1	384	85
TCGA-06-0646	60	male	glioblastoma	G4	WT	5.7496	1	175	90
TCGA-06-0648	77	male	glioblastoma	G4	WT	9.7908	1	298	202
TCGA-06-0649	73	female	glioblastoma	G4	WT	2.1027	1	64	NA
TCGA-08-0246	57	female	glioblastoma	G4	WT	4.1753	1	127	103
TCGA-08-0352	79	male	glioblastoma	G4	WT	1.2822	1	39	NA
TCGA-08-0353	58	male	glioblastoma	G4	WT	8.4164	1	256	164
TCGA-08-0354	52	female	glioblastoma	G4	WT	17.9507	1	546	253
TCGA-08-0355	30	female	glioblastoma	G4	WT	24.5589	1	747	519
TCGA-08-0356	59	female	glioblastoma	G4	WT	31.1014	1	946	447
TCGA-08-0357	49	male	glioblastoma	G4	WT	37.5781	1	1143	155
TCGA-08-0358	50	male	glioblastoma	G4	WT	22.2904	1	678	264
TCGA-08-0359	59	female	glioblastoma	G4	WT	3.3863	1	103	NA
TCGA-08-0360	76	male	glioblastoma	G4	WT	15.3863	1	468	112
TCGA-08-0385	71	male	glioblastoma	G4	WT	2.6959	1	82	NA
TCGA-08-0389	59	male	glioblastoma	G4	WT	15.3534	1	467	NA
TCGA-08-0390	69	male	glioblastoma	G4	WT	13.9633	1	425	NA
TCGA-08-0392	60	male	glioblastoma	G4	WT	0.7233	1	22	NA
TCGA-12-0616	36	female	glioblastoma	G4	WT	14.719	1	448	398
TCGA-DU-6542	25	male	oligoastrocytoma	G3	Mutant	2.4	0	73	NA
TCGA-FG-7643	49	female	oligoastrocytoma	G2	WT	20.0743	0	611	254

Table S2. Slice-level diagnostic performance of the models for the IDH status prediction.

	TCIA Internal Test Set		AHXZ External Test Set	
	**AUC**	**ACC**	**AUC**	**ACC**
		ResNet		
Tumor Slice	0.931	87.9%	0.699	68.4%
Tumor Mask	0.882	84.8%	0.760	70.4%

Tumor + Edema	0.910	87.6%	0.751	72.8%
0.8x Tumor Bbox	0.914	85.5%	0.738	73.9%
1.0x Tumor Bbox	0.929	87.6%	0.748	74.3%
1.2x Tumor Bbox	0.933	89.2%	0.744	73.3%
1.5x Tumor Bbox	0.879	85.6%	0.747	73.6%
average	0.911	86.9%	0.741	72.4%
		Swin Transformer		
Tumor Slice	0.952	91.6%	0.729	71.6%
Tumor Mask	0.928	88.9%	0.788	72.9%
Tumor + Edema	0.898	88.9%	0.781	75.2%
0.8x Tumor Bbox	0.928	87.3%	0.773	76.8%
1.0x Tumor Bbox	0.971	92.7%	0.775	76.5%
1.2x Tumor Bbox	0.975	94.4%	0.765	75.2%
1.5x Tumor Bbox	0.975	93.6%	0.772	76.1%
average	0.947	91.1%	0.769	74.9%
